# Investigation of the Mitigation of DMSO-Induced Cytotoxicity by Hyaluronic Acid following Cryopreservation of Human Nucleus Pulposus Cells

**DOI:** 10.3390/ijms241512289

**Published:** 2023-07-31

**Authors:** Daiki Munesada, Daisuke Sakai, Yoshihiko Nakamura, Jordy Schol, Erika Matsushita, Shota Tamagawa, Kosuke Sako, Shota Ogasawara, Masato Sato, Masahiko Watanabe

**Affiliations:** 1 Department of Orthopedic Surgery, Tokai University School of Medicine, 143 Shimokasuya, Isehara 259-1193, Japan; munesada.daiki.k@tokai.ac.jp (D.M.); jordy.schol@gmail.com (J.S.); s-tamagawa@juntendo.ac.jp (S.T.); k.sako0626@gmail.com (K.S.); ogswr.a.817@gmail.com (S.O.); sato-m@is.icc.u-tokai.ac.jp (M.S.); masahiko@is.icc.u-tokai.ac.jp (M.W.); 2 Center for Musculoskeletal Innovative Research and Advancement (C-MiRA), Tokai University Graduate School, 143 Shimokasuya, Isehara 259-1193, Japan; 3 Research Center for Regenerative Medicine, Tokai University School of Medicine, 143 Shimokasuya, Isehara 259-1193, Japan; kahiko@is.icc.u-tokai.ac.jp (Y.N.); me091130@tsc.u-tokai.ac.jp (E.M.); 4 Department of Medicine for Orthopaedics and Motor Organ, Juntendo University Graduate School of Medicine, 3-1-3 Hongo, Bunkyo-ku 113-8431, Japan

**Keywords:** intervertebral disc, nucleus pulposus, nucleus pulposus progenitor cell, Tie2 receptor, dimethyl sulfoxide, reactive oxygen species, hyaluronic acid, cryopreservation

## Abstract

To develop an off-the-shelf therapeutic product for intervertebral disc (IVD) repair using nucleus pulposus cells (NPCs), it is beneficial to mitigate dimethyl sulfoxide (DMSO)-induced cytotoxicity caused by intracellular reactive oxygen species (ROS). Hyaluronic acid (HA) has been shown to protect chondrocytes against ROS. Therefore, we examined the potential of HA on mitigating DMSO-induced cytotoxicity for the enhancement of NPC therapy. Human NPC cryopreserved in DMSO solutions were thawed, mixed with equal amounts of EDTA-PBS (Group E) or HA (Group H), and incubated for 3–5 h. After incubation, DMSO was removed, and the cells were cultured for 5 days. Thereafter, we examined cell viability, cell proliferation rates, Tie2 positivity (a marker of NP progenitor cells), and the estimated numbers of Tie2 positive cells. Fluorescence intensity of DHE and MitoSOX staining, as indicators for oxidative stress, were evaluated by flow cytometry. Group H showed higher rates of cell proliferation and Tie2 expressing cells with a trend toward suppression of oxidative stress compared to Group E. Thus, HA treatment appears to suppress ROS induced by DMSO. These results highlight the ability of HA to maintain NPC functionalities, suggesting that mixing HA at the time of transplantation may be useful in the development of off-the-shelf NPC products.

## 1. Introduction

Low back pain (LBP) is a common and serious issue that affects people of all ages and can result in significant disability, leading to a considerable socioeconomic burden [1,2]. Although the origin of LBP is complex and often multifactorial [3,4], intervertebral disc (IVD) degeneration is considered one of the primary contributing factors [5,6]. This degenerative process can be precipitated by a variety of aspects, including age, mechanical stress, genetics, and other external stimuli, which may promote an imbalance between anabolic and catabolic processes within the disc. This disparity between anabolic and catabolic processes can lead to a cascade of alterations within the IVD, including biochemical, biomechanical, and inflammatory changes, which further accelerate degeneration. Although the exact mechanism of degeneration is not yet clearly understood, several contributing factors have been identified [7,8,9,10,11]. Unfortunately, the IVD has limited self-repair capacity in part due to its avascular nature [12,13]. Progressive IVD degeneration can lead to a range of serious conditions, including disc herniation, spinal canal stenosis, and degenerative spondylolisthesis. These conditions may cause a variety of symptoms, including lower extremity radicular pain, numbness, muscle weakness, and LBP [14,15], and often lead to poor quality of life [16,17]. Therefore, early intervention for IVD degeneration is important to improve the quality of life of patients.

The standard treatments for discogenic LBP, such as painkillers and physiotherapy, mainly provide temporary relief and do not target the underlying cause. In cases where patients do not respond to these treatments, more invasive procedures like spinal fusion may be considered. However, the effectiveness of these procedures remains controversial and variable [18,19,20]. There is currently a significant medical need for pharmacological or biological therapies that specifically address the degeneration of the IVD to alleviate discogenic pain and prevent further spinal damage. Regenerative medicine has offered novel approaches to target the underlying pathology of IVD degeneration, such as tissue engineering [21,22], growth factor injection [23], and gene therapy [24]. In particular, cell therapy has rapidly advanced [25], with various ongoing clinical trials using different types of cells. These include autologous or allogeneic disc-derived cells and mesenchymal stem cells [26,27,28,29,30,31,32]. Although the most effective cell type is yet to be determined, IVD-derived nucleus pulposus cells (NPC) are a promising option due to their inherent ability to adapt to the harsh IVD environment and produce large amounts of IVD-specific extracellular matrix (ECM) components [28,32].

In the past, our group has identified Tie2 as a marker for human nucleus pulposus (NP) progenitor cells, making NPC possess clonal multipotency and high therapeutic potential for degenerative disc disease [33,34,35,36,37,38,39]. Additionally, we developed a whole-tissue culture method to enable the expansion and maintenance of these Tie2-positive NPC [39]. However, several challenges remain for the adaptation of these progenitor cells as a clinical cell transplantation product, such as methods to produce large amounts of highly potent and safe cells that are ready for transplantation at the time of intervention [40]. The process of freezing and thawing cells is known to have a negative impact on their viability and potential, which may decrease the effectiveness of cell therapy [41,42]. An off-the-shelf product that can be directly transplanted without the need for additional cell culture or removal of cryoprotectants would be ideal for clinical use, accessibility, and reducing costs. Such off-the-shelf cell products are currently undergoing human testing in multiple clinical trials [43,44]. In the mass production of cellular products, a large volume of cells will inevitably be exposed to the cryopreservation medium at harmful temperatures for a certain period of time [45]. It is important to take into account that dimethyl sulfoxide (DMSO) is widely used as a cryoprotectant that is applied to avoid the formation of cytotoxic ice crystals within cells and is suggested as the most commonly applied cryopreservant in the NPC research field [46]. However, research has shown that DMSO harms cells by promoting oxidative stress and disrupting mitochondrial function, which can result in cytotoxicity [47,48,49]. Although it is not yet completely defined how DMSO directly affects human NPC, DMSO-containing media have been shown to be detrimental to NPC viability [45], and studies have also demonstrated that oxidative stress can hinder NPC viability and proliferation potential, encourage cell senescence, and prompt a catabolic phenotype [50]. We have previously shown that the application of the antioxidant N-acetylcysteine (NAC), a reactive oxygen species (ROS) scavenger, could mitigate the damage induced by DMSO-based cryopreservation [47].

Hyaluronic acid (HA), a type of mucopolysaccharide, is present in various parts of the human body, including joint fluid, connective tissue, and the IVD, particularly the NP. Here, HA together with proteoglycans support water retention, reduce biomechanical friction, and prevent excessive loads within cartilage tissues, therefore protecting the joints [51,52]. Moreover, intra-articular injection of HA has been shown to relieve pain associated with knee osteoarthritis by, in part, reducing inflammation [53,54]. HA has therefore been proposed as a promising carrier for cell transplantation in the IVD, as highlighted by several cell transplantation trials [38,55,56]. Furthermore, studies have reported that HA protects mitochondrial DNA from oxidative stress in particular chondrocytes, and contributes to cell survival [57,58]. Based on these findings, we set out to examine the potential of HA to alleviate DMSO-induced oxidative stress for its future application to support and enhance NPC cell therapy products. Specifically, we aim to test whether HA addition can alleviate oxidative stress induced by exposure to DMSO, therefore, enhancing NPC viability and potency.

## 2. Results

### 2.1. Cell Viability and Cell Proliferation Rates

**Figure 1:**
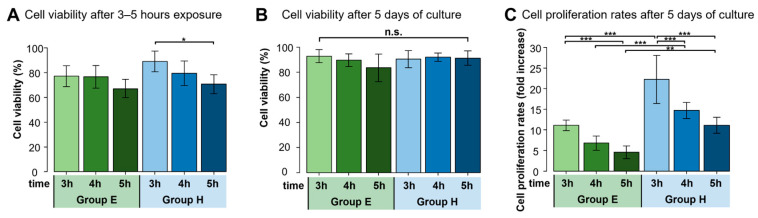
Cell viability after 3–5 h exposure and 5 days of culture, and cell proliferation rates after 5 days of culture. Group E and Group H represent thawed Human NPC suspensions mixed with either 1 mL of A-EDTA or 1 mL of 1% HA, respectively. Indicated time is the number of hours of exposure after mixing. Bars and error bars represent mean and standard deviation, respectively. (**A**) Cell viability after 3–5 h exposure was not significantly different at each exposure time. (*n* = 7, * *p* < 0.05) (**B**) Cell viability after 5 days of culture was not significantly different at each exposure time. (*n* = 7, n.s. not significant) (**C**) Cell proliferation rates after 5 days of culture were significantly higher in Group H than in Group E. (*n* = 7, ** *p* < 0.01, *** *p* < 0.001).

**Figure 2:**
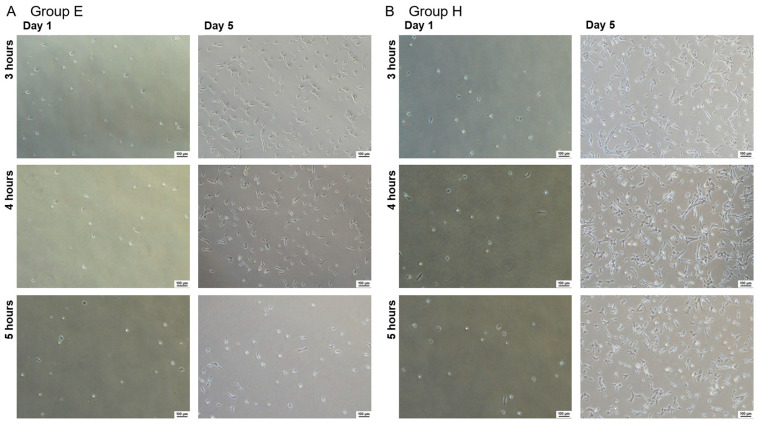
Representative images of cell morphology and proliferation following 3-, 4-, or 5-h exposure in DMSO-containing media and subsequent culture. Images taken at 1 and 5 days. (Scale bars = 100 μm). Group E and Group H represent thawed Human NPC suspensions mixed with either 1 mL of A-EDTA or 1 mL of 1% HA, respectively. Indicated time is the number of hours of exposure after mixing. (**A**) were Images of Group E, (**B**) were Images of Group H.

Human NPC suspensions derived from a cryopreserved condition were thawed and mixed with either 1 mL of albumin-containing EDTA-PBS (A-EDTA) (Group E) or 1 mL of 1% HA (Group H) and were incubated for 3, 4, or 5 h at room temperature. We found no significant differences in cell viability following 3–5 h of DMSO exposure between the two groups (Figure 1A). However, while there was no significant difference in cell viability after 5 days of culture (Figure 1B), there was a significant difference in cell proliferation rate. Specifically, the cell proliferation rate was approximately 11.1 ± 1.3-fold in Group E after 3-h DMSO exposure, compared to a 22.3 ± 5.8-fold increase for Group H with the same exposure time, a two-fold increase (*p* < 0.001). Although the proliferation rate decreased with an increase in DMSO exposure time, a similar 2-fold difference was consistently observed between Group E and H at each incubation length (Figure 1C). Inverted phase contrast microscopy confirmed these trends (Figure 2).

### 2.2. Tie2 Positivity Rates and Tie2 Positive Cells Numbers

**Figure 3:**
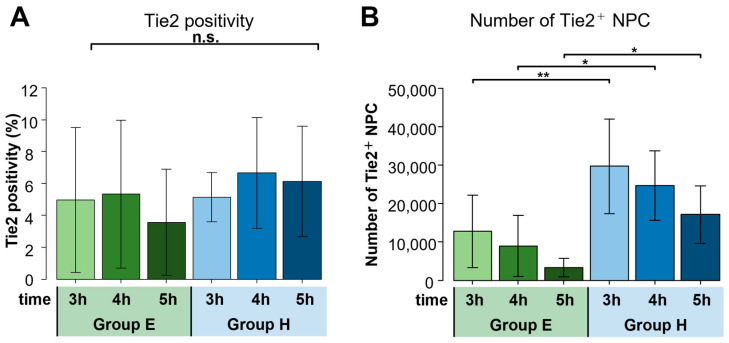
Tie2 positivity rates and Tie2 positive (Tie2^+^) cells number. Group E and Group H represent thawed Human NPC suspensions mixed with either 1 mL of A-EDTA or 1 mL of 1% HA, respectively. Indicated time is the number of hours of exposure after mixing. Bars and error bars represent mean and standard deviation, respectively. (**A**) Tie2 positive rates showed no significant differences between the two groups after 5 days of culture. (*n* = 7, n.s. not significant) (**B**) The number of Tie2^+^ NPC was significantly higher in Group H compared to Group E. (*n* = 7, * *p* < 0.05, ** *p* < 0.01).

Harvested NPC following 5 days of culture were analyzed through flow cytometry for the number and rate of Tie2 positive cells. Here we found no significant difference in the rate of Tie2 positivity between the two groups, regardless of DMSO exposure times (Figure 3A). However, a significant difference was observed in the overall number of Tie2 positive cells after 5 days of culture, favoring Group H compared to Group E. Moreover, both groups showed a decline in Tie2-positive NPC numbers with an increase in DMSO exposure times (Figure 3B).

### 2.3. The Intracellular and Mitochondrial ROS of NPC

**Figure 4:**
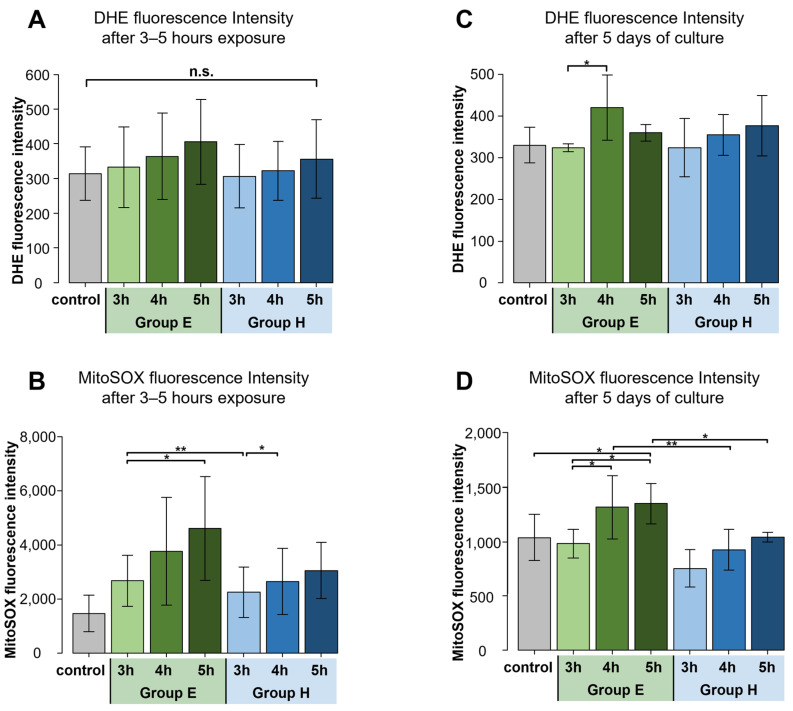
Quantitative analysis of mean DHE and MitoSOX fluorescence intensity. Group E and Group H represent thawed Human NPC suspensions mixed with either 1 mL of A-EDTA or 1 mL of 1% HA, respectively. Indicated time is the number of hours of exposure after mixing. Bars and error bars represent mean and standard deviation, respectively. (**A**) DHE fluorescence intensity after DMSO exposure showed no significant differences between the two groups but tended to increase with longer exposure to DMSO. (*n* = 7, n.s. not significant) (**B**) MitoSOX fluorescence intensity after 3 h exposure showed significant differences between the two groups and tended to increase with longer exposure to DMSO. (*n* = 7, * *p* < 0.05, ** *p* < 0.01) (**C**) DHE fluorescence intensity after 5 days of culture showed no significant differences between the two groups. (*n* = 7, * *p* < 0.05) (**D**) MitoSOX fluorescence intensity after 5 days of culture showed significantly lower in Group H than in Group E. (*n* = 7, * *p* < 0.05, ** *p* < 0.01).

Next, we examined mitochondrial superoxide levels via dihydroethidium (DHE) and MitoSOX labeling following exposure to DMSO. As a control, NPC samples directly after thawing were spun down and DMSO-containing media was removed before seeding and culturing. These cells were thus minimally subjected to DMSO. The intracellular- and mitochondrial superoxide tended to increase with longer exposure to DMSO, a trend more evident within Group E (Figure 4A,B). Subsequent culture of the DMSO-exposed NPC showed in particular a significant increase in mitochondrial superoxide levels after 5 days of culture for Group E samples, which were significantly higher than Group H (Figure 4D). DHE levels showed less evident differences (Figure 4C).

## 3. Discussion

To summarize the results of this study, mixing HA after freeze-thawing maintained the proliferation of NPC, which in turn could increase the yield of Tie2-positive NPC. Moreover, HA was able to suppress ROS production following DMSO exposure.

DMSO remains the cryopreservation agent of choice according to a most recent consensus paper from the spine research field [46]. Nevertheless, high concentrations of DMSO (≥10% *v*/*v*) are known to induce cytotoxicity by increasing intracellular DMSO concentrations through the formation of cell membrane pores and increasing ROS levels in a concentration-dependent manner [48]. Even at 5–10% DMSO concentrations, which are commonly found in most cryopreservation media, DMSO has been found to promote ROS production and apoptosis, as well as decrease overall cell viability and potency [40,45,47]. Although we have previously shown that the transplantation of human NPC directly from a cryopreserved state can be an effective and safe strategy to restore induced disc degeneration [38], concerns remain on the impact of DMSO on the transplanted cells as well as the endemic cells within the IVD. Reporting from our previous clinical trial has indicated that processing off-the-shelf NPC from thawing in the cell processing center to the final injection took an average of 122.9 min, subjecting the cell products to DMSO-containing media during these procedures [35,45]. Furthermore, due to the limited diffusion of the disc in part owing to the disc’s avascular nature [12,13,59], DMSO may induce oxidative stress locally for several hours after transplantation. This further emphasizes the need to limit the impact of DMSO to optimize the regenerative potential of cell therapeutic agents.

Therefore, it is likely beneficial to shorten the exposure time or limit the impact of DMSO to optimize the regenerative agent. One method to solve this issue is by removing the cryopreservation solution before transplantation. This can easily be done by simple centrifugation and subsequent washing of the samples; however, this may increase the cost, time, and medical staff burden of the procedure. Therefore, the removal of the cryopreservation solution could be a hurdle for the successful commercialization of cell transplantation products. However, a previous canine study has shown the potential of direct off-the-shelf cell transplantation, without any clear adverse events [38]. Whether or not removing cryopreservation solution remains a matter of further study.

HA and its derivatives have important medical and industrial applications [51]. It has been employed in multiple areas for its anti-inflammatory characteristic [51,60], pain-relieving potential [61,62,63], and as a direct lubricant [54]. Moreover, it has been shown that HA exhibits antioxidant properties for articular chondrocytes [57,58,64]. Reports have shown that levels of H_2_O_2_ and O_2_^−^, known ROSs, are lower in the synovial fluid following intra-articular injection of HA compared to the before-treatment levels. Moreover, cell death can be rescued by adding HA to chondrocytes exposed to various concentrations of H_2_O_2_ [64]. HA antioxidant properties have similarly been shown in other cell types [65,66,67,68]. Therefore, we set out to determine if HA could limit the oxidative stress in NPC, resulting from DMSO, to increase the potency of a potential off-the-shelf transplantation product. Here, we mimicked a clinical setting in vitro by subjecting the cells to direct encapsulation in HA immediately after thawing without removing DMSO followed by incubation for several hours before culture. The results of this study showed that ROS levels after incubation were higher than those after 5 days of culture, and this was especially true regarding mitochondrial ROS. This suggests that the effects of the DMSO-based freezing/thawing procedure were still affecting the NPC after the 5-day culture, as measured ROS intensity patterns showed similar trends to those observed before cell seeding. This underlines the potential impact of this cell processing aspect on the long-term potency of cell transplantation products. Moreover, we showed that the addition of HA alleviated oxidative stress, and proliferative capacity was maintained, even in cells exposed to DMSO, without reducing Tie2 expression levels. As a result, the yield of Tie2-positive NPCs increased. Tie2, a tyrosine kinase receptor with angiopoietin-1 as a ligand, is a marker for NP progenitor cells [33]. NPCs with high Tie2 expression have been shown to possess a strong capacity to produce IVD-specific ECM components, such as type II collagen and aggrecan [37,39]. Moreover, these cells present an overall high regenerative and differentiation capacity [69]. They are thus expected to be a potent regenerative cell population for IVD repair [32], and as such multiple attempts have been made to enhance the yield or expansion of these NP progenitor cells [39,70,71,72,73]. Here, we could show that the recovery from DMSO-induced cytotoxicity through HA exposure, can enhance Tie2 expressing NPC yields, therefore offering a promising and easy adoption to enhance the development of NP progenitor cell-based transplantation products.

Despite our promising results, some limitations should be considered. In this study, we did not evaluate the ECM production capacity of the resulting cells. In addition, our experiments were conducted only in vitro, and it is unclear how DMSO and HA will impact cells in the harsher IVD upon transplantation. Furthermore, since the mechanism by which the addition of HA reduces the oxidative stress of DMSO has not been yet elucidated, we are now considering examining the effects of neutralizing CD44, a known receptor for HA, and confirming the impact on our observed beneficial effects [58,64].

Moreover, additional care will be required for the method of transplantation. It has been reported that misplacement or leakage of transplanted cells from the IVD to the surrounding tissues may result in the formation of undesirable tissue formation e.g., osteophytes [74]. Therefore, it could be useful to prevent osteophyte formation that cellular MR imaging for tracking transplanted cells [75], and a technique for non-invasive monitoring of minimally invasive stem delivery into the vertebral disc [76]. Here HA is also likely to support cell transplantation, by forming an adhesive carrier supporting the retention of the de novo cells within the disc space.

## 4. Materials and Methods

### 4.1. Human NP Cell Isolation and Culture

The study received approval from the Institutional Review Board for Clinical Research at Tokai University (application number: 17R-173), indicating that all research procedures described in the study met ethical and safety standards established by our institution. The study involved the collection of human IVD tissue samples from 7 patients (mean age ± standard deviation, 17.6 ± 2.7 years) who underwent surgery for lumbar disc herniation at Tokai University Hospital and related facilities (Table 1). Before tissue collection, all patients provided informed written consent, indicating their consent for the use of surgical waste for research. Alternatively, for patients under the age of 18, informed consent was obtained from their parent (s) or legal guardian (s).

NP cells were isolated and cultured using previously described methods [37,39]. The collected surgical NP tissue was first washed with saline and then cut into 3–5 mm diameter pieces using scissors and scalpels [37]. The culture conditions were designed to mimic the culture conditions of a cell transplantation product under development, as outlined in the work of Sako et al. [39]. NP fragments were added directly to a complete culture medium consisting of Dulbecco’s modified Eagle’s medium (DMEM; Gibco, Grand Island, NY, USA) and α-minimal essential medium (αMEM; Gibco, Grand Island, NY, USA) supplemented with 20% (*v*/*v*) fetal bovine serum (FBS; Sigma-Aldrich, St. Louis, MO, USA) and 1% penicillin/streptomycin (Gibco, Grand Island, NY, USA). The tissue fragments were then cultured in polystyrene 6-well plates (IWAKI, Tokyo, Japan) with approximately 0.3 g of NP tissue seeded per 3 mL culture medium in a single well. The fragments were cultured for 14 days at 37 °C in 5% CO_2_ and 5% O_2_ without media replenishment.

After two weeks, tissue fragment suspensions were collected, centrifuged, and the supernatant was discarded. The tissue was then resuspended in 10 mL of TrypLE Express (Thermo Fisher Scientific, Tokyo, Japan) and digested at 37 °C for 30 min under gentle swirling. The resulting suspension was again collected, centrifuged, and subsequently digested for 2 h at 37 °C using 10 mL of αMEM supplemented with 10% (*v*/*v*) FBS and 0.25 mg/mL collagenase P (Roche, Basel, Switzerland). After digestion, the suspension was filtered using a 40 μm cell strainer (Corning, NY, USA), centrifuged, and the supernatant was removed. The resulting cells were then seeded at a density of 3.0 × 10^4^ cells per well in 100-mm dishes (Corning) and cultured in a medium as previously specified for 7 days without any media change. IVD-derived cells were used after the third passage.

NPCs were treated with 5 mL of TrypLE Express for 3 min and then collected into a 15 mL conical tube. Samples were centrifuged at 1200 rpm for 5 min at 4 °C, resuspended in 5 mL of buffered saline, and centrifuged again at 1200 rpm for 5 min at 4 °C. After collecting the cultured cells, they were aliquoted into a cryotube at 3.0 × 10^5^ cells in 1 mL of CryoStor^®^ CS10 (CS10) (STEMCELL Technologies, Vancouver, BC, Canada), which contains 10% DMSO, and the samples were cryopreserved in stages to −80 °C using a controlled-rate cryopreservation device (Bicell, Nihon Freezer, Tokyo, Japan). The next day, the samples were stored in a liquid nitrogen container at −196 °C. The cryopreservation period was set to 2 weeks.

### 4.2. Incubation and Culture for Transplantation Simulation

**Figure 5:**
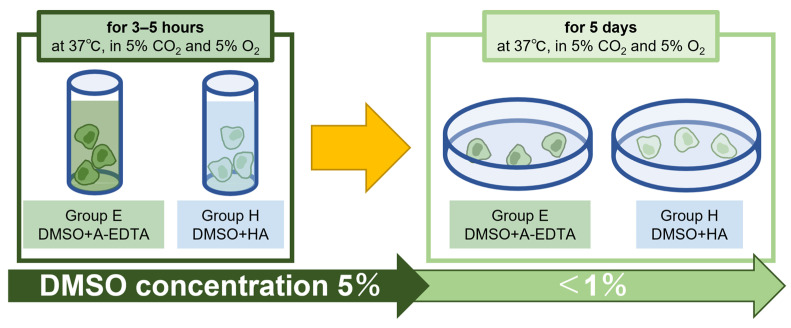
Simulation of NPC transplantation in degenerating IVD in vitro. Cryopreservation solution was not removed from thawed NPCs and mixed in equal volumes with 1 mL each of A-EDTA (Group E) or ARTZ^®^ (1% HA solution) (Group H); after 3–5 h of incubation, DMSO was removed as if diffusion metabolism occurred in IVD (DMSO concentration less than 1%) and cultured for 5 days.

Conditions were chosen to mimic the environment transplanted cells would be subjected to in the IVD i.e., low nutrition and low oxygen levels. Although DMSO concentration is expected to thin out after transplantation due to diffusion, this experiment was set up in a more severe environment where DMSO concentrations did not thin out, until DMSO was removed. Frozen NPC solutions were thawed slowly in a water bath set to 37 °C for about one minute. When fully defrosted, the 1 mL samples were transferred 15 mL conical tube and either mixed with 1 mL of A-EDTA forming Group E or mixed with 1 mL of ARTZ Dispo^®^ (Seikagaku Corporation, Tokyo, Japan) forming Group H. ARTZ Dispo^®^ contains 1% sodium hyaluronate solution with an average molecular weight range of 5.0 × 10^5^ to 1.2 × 10^6^ Daltons, at a concentration of 25 mg/2.5 mL and is being applied as an effective intra-articular agent for knee OA [77,78]. The solutions were carefully mixed by pipetting and kept with the lids slightly open (for gas exchange) and incubated for 3, 4, or 5 h at 37 °C in a 5% CO_2_ and 5% O_2_ incubator. After incubation, NPC samples were spun down and DMSO-containing media was removed. The NPCs were resuspended and seeded at 3.0 × 10^4^ cells per 100-mm dishes in 6 mL αMEM supplemented with 30% FBS, 10 ng/mL basic fibroblast growth factor (FGF2; PeproTech, Cranbury, NJ, USA), and 1% penicillin/streptomycin, and cultured for 5 days at 37 °C in 5% CO_2_ and 5% O_2_ (Figure 5). The cultured NPCs were observed, and images were captured using an inverted phase contrast microscope at 1- and 5-days of culture.

### 4.3. Cell Viability and Cell Proliferation Rates

The cells were exposed for 3, 4, and 5 h and were collected, spun down (to remove medium), and suspended in 1 mL of A-EDTA. On the other hand, the cells cultured for 5 days were treated with 5 mL of TrypLE Express for 3 min and collected, and the medium was removed and mixed using the same method. The number of viable and non-viable cells was determined using the trypan blue exclusion method. The cell proliferation rates were calculated by dividing the total number of cells after culture by the number of cells seeded before culture (3.0 × 10^4^ cells).

### 4.4. Flow Cytometry Analysis

NPCs were examined using a FACS Calibur flow cytometer (BD Biosciences, Franklin Lakes, NJ, USA), following procedures described in a previous study [37]. Living cells were selectively analyzed by implementing a propidium iodide-negative gate. NPC was examined using flow cytometry to determine the proportion of cells expressing NP progenitor cell marker Tie2 [33,37]. NPCs were comparatively analyzed to isotype control antibodies, as previously stated in Sakai et al. [37]. The number of Tie2-positive NPC was estimated by multiplying the number of living cells counted at 5 days of culture by the Tie2-positive rates measured by flow cytometry.

### 4.5. Measurement of Intracellular and Mitochondrial ROS

NPC samples following either 3–5 h DMSO exposure or following 5 days of culture, were collected, washed twice in phosphate-buffered saline (PBS), and counted. A total of 3.0 × 10^4^ NPC per condition were incubated with 10 μM dihydroethidium (DHE; Invitrogen) or 5 μM MitoSOX Red (Invitrogen) for 30 min in the dark at 37 °C. NPC samples that were directly spun after thawing to quickly remove DMSO-containing media were removed and were seeded and cultured, and functioned as a control. The levels of intracellular and mitochondrial superoxide were analyzed using FACS. The mean fluorescence intensity of DHE and MitoSOX was determined through an excitation wavelength of 488 nm and the measurement of the emission at 578 nm wavelength [47].

### 4.6. Statistical Analysis

All statistical analyses were performed with Easy R (EZR; Saitama Medical Center, Jichi Medical University, Saitama, Japan, version 1.61), which is a graphical user interface for R (The R Foundation for Statistical Computing, Vienna, Austria, version 4.2.2). More precisely, it is a modified version of R commander (version 2.8-0) designed to add statistical functions frequently used in biostatistics [79]. All values are presented as mean (±standard deviation) unless specifically stated otherwise. Statistical differences were determined via one-way ANOVA, followed by Dunnett’s multiple comparison or Tukey’s comparison test. A *p*-value of <0.05 was considered statistically significant.

## 5. Conclusions

The addition of HA to thawed cryopreservation medium helped to mitigate the cytotoxicity of DMSO, and thus enhanced NPC proliferation. Moreover, HA was able to increase the number of harvestable Tie2-positive NPCs. These results suggest the ability of HA to maintain NPC functionalities. Therefore, mixing HA at the time of transplantation may contribute to the enhancement of cell transplantation products for degenerative disc disease.

## Figures and Tables

**Table 1:** Details of clinical samples. Age, sex, total weight of IVD tissue collected at the time of surgery, and weight of NP tissue after selection are listed for each sample. LDH: lumber disc hernia, IVD: intervertebral disc, NP: nucleus pulposus.

Code	Age (Years)	Sex	Pathology	IVD Tissue (g)	NP Tissue (g)
A18	18	M	LDH	5.88	3.73
T16	16	M	LDH	2.03	2.03
A14	14	F	LDH	1.74	1.54
A16	16	M	LDH	1.81	1.5
T19	19	M	LDH	8.05	7.59
A17	17	M	LDH	4.25	2.99
T23	23	M	LDH	2.03	1.03

## Data Availability

Data can be requested from the corresponding authors upon reasonable request.
